# Synthesis and In Vitro Study of a Dual-Mode Probe Targeting Integrin α_v_β_3_

**DOI:** 10.1186/s11671-018-2695-y

**Published:** 2018-09-11

**Authors:** Yali Zhang, Xuna Zhu, Lidong Liu, Sen Hong, Zhichao Zuo, Peng Wang, Danke Su

**Affiliations:** Departments of Radiology, Affiliated Tumor Hospital of Guangxi Medical University, 71 Hedi Road, Nanning, 530021 Guangxi Zhuang Autonomous Region People’s Republic of China

**Keywords:** Malignant tumors, Integrin α_v_β_3_, Molecular imaging, Dual-mode molecular probe, Visual monitoring, Tumor metastasis

## Abstract

Malignant tumors constitute a serious disease that threaten human life, and early diagnosis and metastasis prediction are critical to the choice of treatment plan and the timing of treatment. Integrin α_v_β_3_, which has received broad attention as a molecular marker of the tumor neovasculature, is an important target for monitoring tumorigenesis and progression in molecular imaging research. This study reports a magnetic resonance (MR)/fluorescence dual-mode molecular probe, cRGD-Gd-Cy5.5, which targets the integrin α_v_β_3_ receptor and uses liposomes as carrier. The obtained nanoprobe had a size of 60.08 ± 0.45 nm, with good dispersion in water, a uniform distribution of sizes, desirable stability, and high relaxivity. Its r1 relaxation rate was 10.515 mM^−1^ s^−1^, much higher than that of other Gd chelates in clinical use. The probe showed no cytotoxicity at the tested concentrations in vitro, and its ability to target A549 cells and SUNE-1-5-8F cells was preliminarily evaluated through in vitro fluorescence imaging and MR imaging. The results demonstrated that the cRGD-Gd-Cy5.5 nanoprobe had good characteristics, showing desirable stability and biosafety, a high T1 relaxation rate, and strong targeting and binding to tumors with high expression of integrin α_v_β_3_. Therefore, cRGD-Gd-Cy5.5 is a promising agent for the visual monitoring of tumor metastasis.

## Background

Malignant tumor infiltration and distant metastasis constitute the main cause of treatment failure. The process of malignant tumor metastasis requires the destruction of the extracellular matrix (ECM) and the tumor neovasculature [1]. Therefore, in vitro noninvasive monitoring of molecular markers related to tumor neovascularization is especially crucial in the prediction of tumor metastasis and early diagnosis. Integrin α_v_β_3_, a widely studied molecular marker of the tumor neovasculature, has received extensive attention. This integrin is closely associated with the adhesion, proliferation, and differentiation of vascular endothelial cells during tumor neovascularization [2].

The progress in molecular imaging has enabled noninvasive monitoring of tumor growth at the molecular level. Molecular imaging studies of the target molecules of interest must have two basic elements: (1) suitable targeting components and (2) signal components that can be detected by imaging techniques. Integrin α_v_β_3_, which specifically recognizes and binds to the arginine–glycine–aspartate (Arg-Gly-Asp, RGD) sequence in ECM proteins, is activated through conformational changes. Thus, the RGD sequence has been widely used in the synthesis of tumor-targeting tracers as an important targeting component [3]. Magnetic resonance (MR) imaging has become one of the most powerful molecular imaging diagnostic tools for monitoring tumors because of its non-ionizing radiation, high soft tissue resolution, and non-limiting penetration depth [4, 5]. Statistical analysis indicated that approximately 50% of MR examinations require the use of contrast agents to improve the quality of the images [6, 7]. Near-infrared fluorescence imaging is a type of optical imaging, and the wavelength of near-infrared light, which is the earliest discovered non-visible light, ranges from 700 to 900 nm. Near-infrared fluorescence imaging techniques allow surgeons to visualize, delineate, and resect tumors during surgeries [8, 9]. However, with changes in the human disease spectrum, single-mode molecular imaging fails to meet the requirements of precision diagnosis because of its high false-positive and false-negative rates. Thus, molecular imaging techniques based on the integration of multiple modes will be a new trend in the development of molecular imaging [10].

**Scheme 1:**
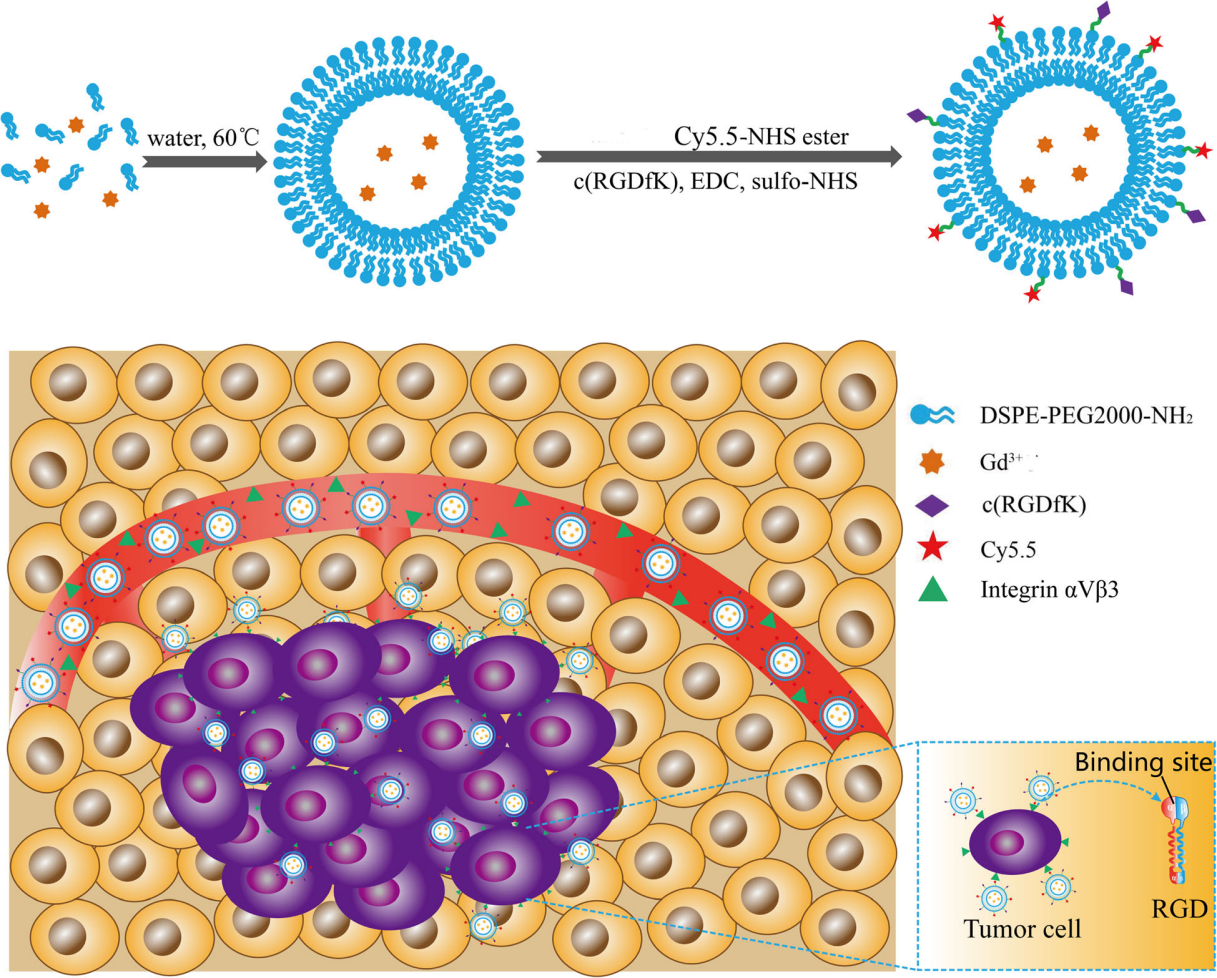
The synthetic route of the Gd-doped liposomes, followed by RGD conjugation and Cy5.5 for bio-applications, for highly sensitive detection of integrin α_v_β_3_

In this study, we prepared a dual-mode molecular probe cRGD-Gd-Cy5.5 that can be used in the histological and functional imaging of tumors (Scheme 1). Compared with the molecular probes with Fe_3_O_4_ as the signal unit [11, 12], cRGD-Gd-Cy5.5 was outstanding with (a) high paramagnetism (seven unpaired electrons around Gd^3 +^); (b) ability to bind with the carrier to form a stable chelation agent, such as Magnevist widely used in clinic; (c) high space resolution, and higher image definition than negative contrast agents [13, 14].We selected liposomes as the carrier molecule that links the MR contrast agent to the fluorescence contrast agent. Common phospholipids have two long hydrophobic hydrocarbon chains and a hydrophilic group in their molecular structures. When added to water or buffer solution, appropriate amounts of phospholipid molecules assume arrangements in specific directions, with their hydrophilic groups facing the water phase on both sides and the hydrophobic hydrocarbon chains facing each other to form the bilayer in liposomes. RGD-targeting cyclic peptides and Cy5.5 fluorescent molecules were conjugated to the liposome surface through an introduced polyethylene glycol (PEG) phospholipid with amino groups, and finally, Gd ions were encapsulated in the liposomes to achieve the construction of the targeted multimodal molecular probe with desirable characteristics in terms of structure, composition, size, shape, stability, and MR relaxivity. Its cytocompatibility was assessed through a cytotoxicity assay and cell morphology observation. Finally, the potential of cRGD-Gd-Cy5.5 for T1-weighted MR imaging and fluorescence imaging of in vitro cancer cells was assessed.

## Results

### Characterization of the cRGD-Gd-Cy5.5 Nanoprobe

**Fig. 1:**
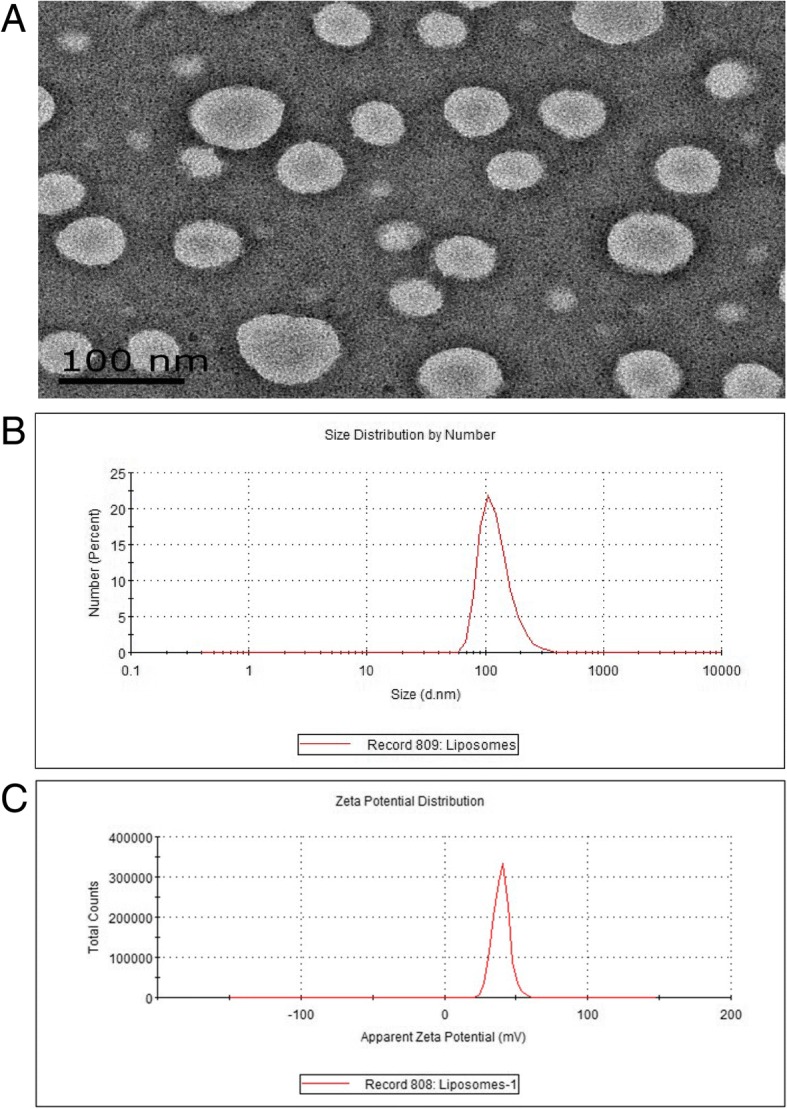
Characterization data of CRGD-GD-CY5.5 nanoprobe. **a** Low magnification TEM images of cRGD-Gd-Cy5.5. **b** The particle size was detected by size analysis, and the average size of the probe was 124.2 nm. **c** Zeta potential is about 39.5 mV, the apparent surface is electropositive

The cRGD-Gd-Cy5.5 solution was a pale pink clear liquid, with no obvious precipitation after standing for a while, indicating good dispersion. Transmission electron microscopy (TEM) revealed the appearance of the cRGD-Gd-Cy5.5 liposomal complex as a spherical shape of uniform size, with no granule aggregation or damage after negative staining with 2% sodium phosphotungstate, as shown in Fig. 1a. The particle size distribution was consistent, ranging from 50 to 80 nm, with an average particle size of 60.08 ± 0.45 nm. The hydrated particle size of the cRGD-Gd-Cy5.5 liposomal complex measured via dynamic light scattering (DLS) was 124.2 ± 0.215 nm (Fig. 1b). As shown in the figure, the nanoparticles exhibited a narrow size distribution in water and good dispersion. The surface zeta potential was 39.5 ± 1.65 mV, showing a positive surface charge, which is consistent with the presence of amino groups on the surface (Fig. 1c).

**Fig. 2:**
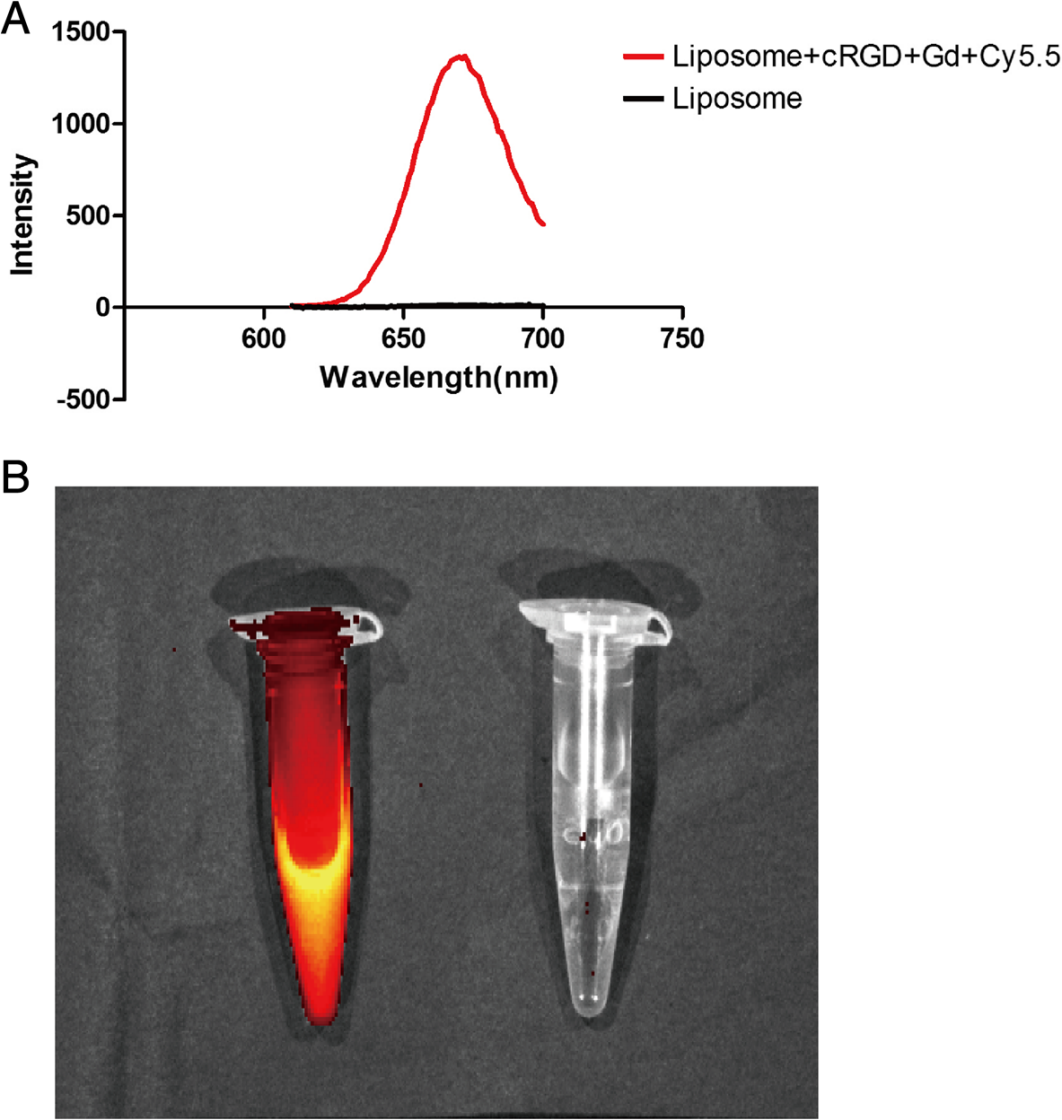
Fluorescence properties of cRGD-Gd-Cy5.5 nanoprobe. **a** The liposome labeled with fluorescence Cy5.5 was detected by multifunctional enzyme analyzer, and there was a visible emission peak at 670 nm at 600-nm excitation light. **b** Under 600-nm excitation light, the liposome-coupled fluorescent Cy5.5 (left) has an obvious luminescence, while the blank liposome (right) does not have fluorescence imaging

Multifunctional microplate assay showed that the blank liposomes do not have UV absorption region and liposomes coupled with fluorescent Cy5.5 in 600 nm excitation light have obvious absorption peak at 685 nm (Fig. 1a), consistent with the emission wavelength of fluorescent Cy5.5. Fluorescent molecules showed that Cy5.5 was successfully coupled. The results of the small animal fluorescence imaging system showed that under the 600-nm excitation light, the liposome-conjugated fluorescent Cy5.5 had obvious red fluorescence, while the blank liposome had no fluorescence signal (Fig. 2b).

### MR Relaxometry of the cRGD-Gd-Cy5.5 Nanoprobe

**Fig. 3:**
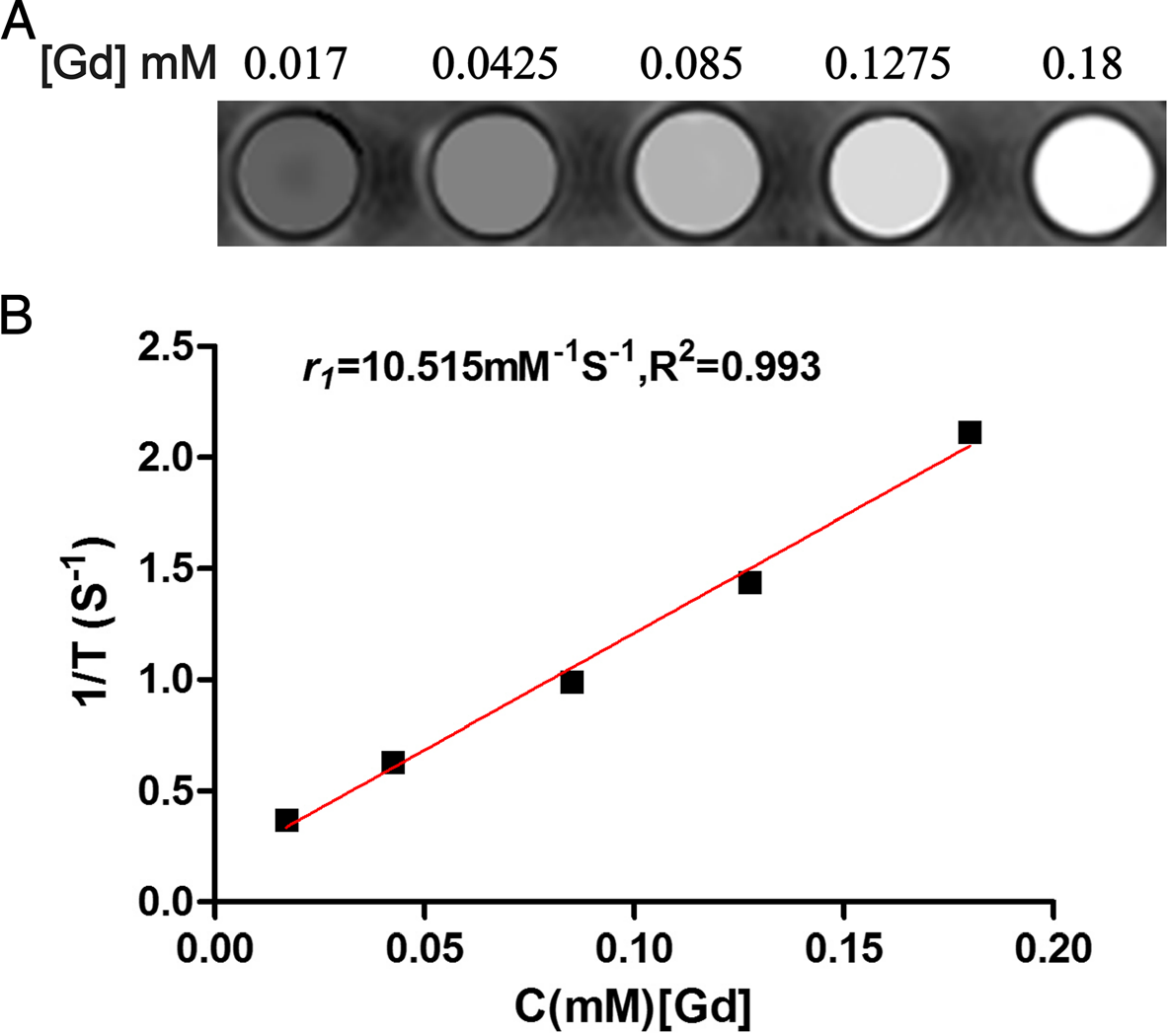
The relaxation properties of cRGD-Gd-Cy5.5 nanoprobes. **a** T1**-**weighted images (Siemens, Verio,3.0T, MOLLI, sequence: TR = 5.8 ms, TE = 3.66 ms, TI = 16–3200 ms) of these particles at different concentrations (Gd). **b** The fitting curves of Gd concentration and relaxation time; the r1 value of cRGD-Gd-Cy5.5 was 10.515 mM^−1^s^−1^

The r1 relaxation rate is an important parameter for evaluating the imaging performance of T1 MR contrast agents. Therefore, we measured the T1 relaxation time of the probe cRGD-Gd-Cy5.5 with different Gd concentrations. As shown in Fig. 3b, the r1 relaxation rate of cRGD-Gd-Cy5.5 was 10.515 mM^−1^ s^−1^ after linear fitting, much higher than that of the clinical product Magnevist (4.56 mM^−1^ s^−1^). The high r1 relaxation rate of cRGD-Gd-Cy5.5 may be the result of the special spatial structure of the carrier liposomes and the biological signal amplification effect of the liposomes. As the Gd concentration increased, cRGD-Gd-Cy5.5 enhanced the MR signal intensity (Fig. 3a). These results demonstrated that the synthetic cRGD-Gd-Cy5.5 meets the needs for high image contrast in MR imaging.

### Cytotoxicity Study of the cRGD-Gd-Cy5.5 Nanoprobe

**Fig. 4:**
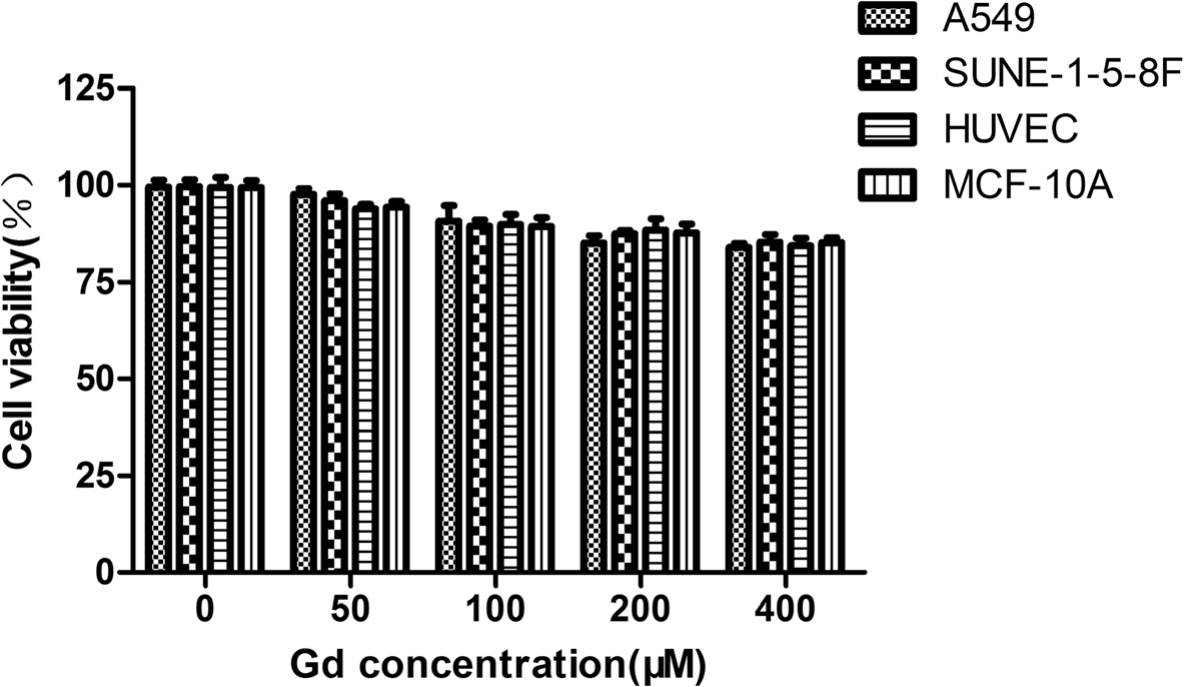
Cytotoxicity test. Cell viability of human lung adenocarcinoma (A549), nasopharyngeal carcinoma cell line (SUNE-1-5-8F), human umbilical vein endothelial cell (HUVEC), and breast normal cell line (MCF10A) incubated with different concentrations of cRGD-Gd-Cy5.5 for 24 h

The in vitro cytotoxicity of cRGD-Gd-Cy5.5 was assessed through the CCK-8 assay, with comparison to the viability of cells in the untreated group (100%). All the cells showed cell viability greater than 70% within the range of tested Gd concentrations (50–400 μM) (Fig. 4), indicating that this molecular probe has good cell compatibility. Notably, the cell viability was still higher than 70% even after 24 h of incubation with 400 μM Gd, a concentration substantially higher than the dosages used in vitro and in vivo.

### Immunofluorescence Staining and Flow Cytometry Assay of Integrin αvβ3

**Fig. 5:**
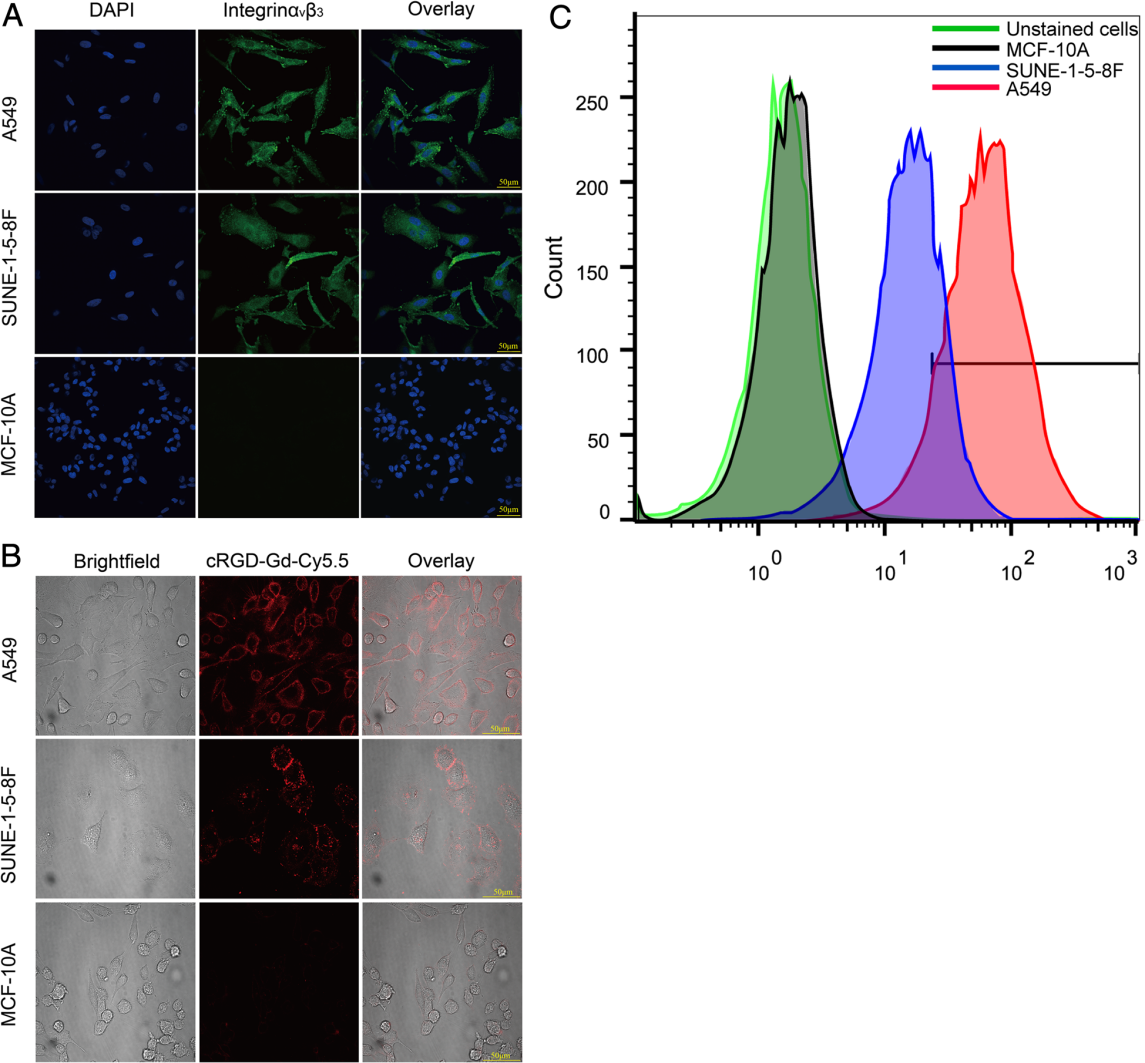
Confocal laser scanning microscopy (CLSM) images of A549, SUNE-1-5-8F, and MCF-10A cells. **a** Cellular immunofluorescence images of integrin αvβ3. A549 and SUNE-1-5-8F cell line expressed in the cell membrane, MCF-10A cells almost no expression of integrin α_v_β_3_. **b** A549, SUNE-1-5-8F, and MCF10A cells incubated with cRGD-Gd-Cy5.5 at 200 μg mL^−1^ concentration at 37 °C for 1 h. The amount of probes bound to cells agrees with the results of cellular immunofluorescence. **c** Flow cytometry assay for detecting the integrin αvβ3 expressions in the A549, SUNE-1-5-8F, and MCF-10A cells

The results of immunofluorescence showed that the integrin αvβ3 expression in A549 and 5-8F cells was localized in the cell membrane and cytoplasm. The integrin of A549 cells was mainly located in the cell membrane, and the fluorescence intensity on the surface of A549 cells was higher than that of SUNE-1-5-8F cells. The fluorescence intensity of MCF-10A cells was extremely weak, demonstrating that nearly no integrin αvβ3 was expressed on the surface of mammary epithelial cells (Fig. 5a). The flow cytometry results are shown in Fig. 5c. The integrin αvβ3 expression levels rank as follows: A549 (89.07%) > SUNE-1-5-8F (63.84%) > MCF-10A (1.56%), indicating the αvβ3 levels in cancer cells were significantly higher than those in normal mammary gland cells.

### Cellular Uptake of cRGD-Gd-Cy5.5

Based on the results of the immunofluorescence staining, A549 and 5-8F cells incubated with cRGD-Gd-Cy5.5 were used as the experimental groups, while the MCF-10A cells incubated with cRGD-Gd-Cy5.5 were employed as the control group. After incubation with cRGD-Gd-Cy5.5, red fluorescence signals were observed in the membrane of both A549 and 5-8F cells, with the signal intensity in A549 cells higher than that in SUNE-1-5-8F cells (Fig. 5b). This result is consistent with the expression of integrin αvβ3 detected by immunofluorescence staining. Nearly no red fluorescence signal was found in the membrane of the MCF-10A cells in the control group.

### In Vitro MR Imaging and Fluorescence Imaging

**Fig. 6:**
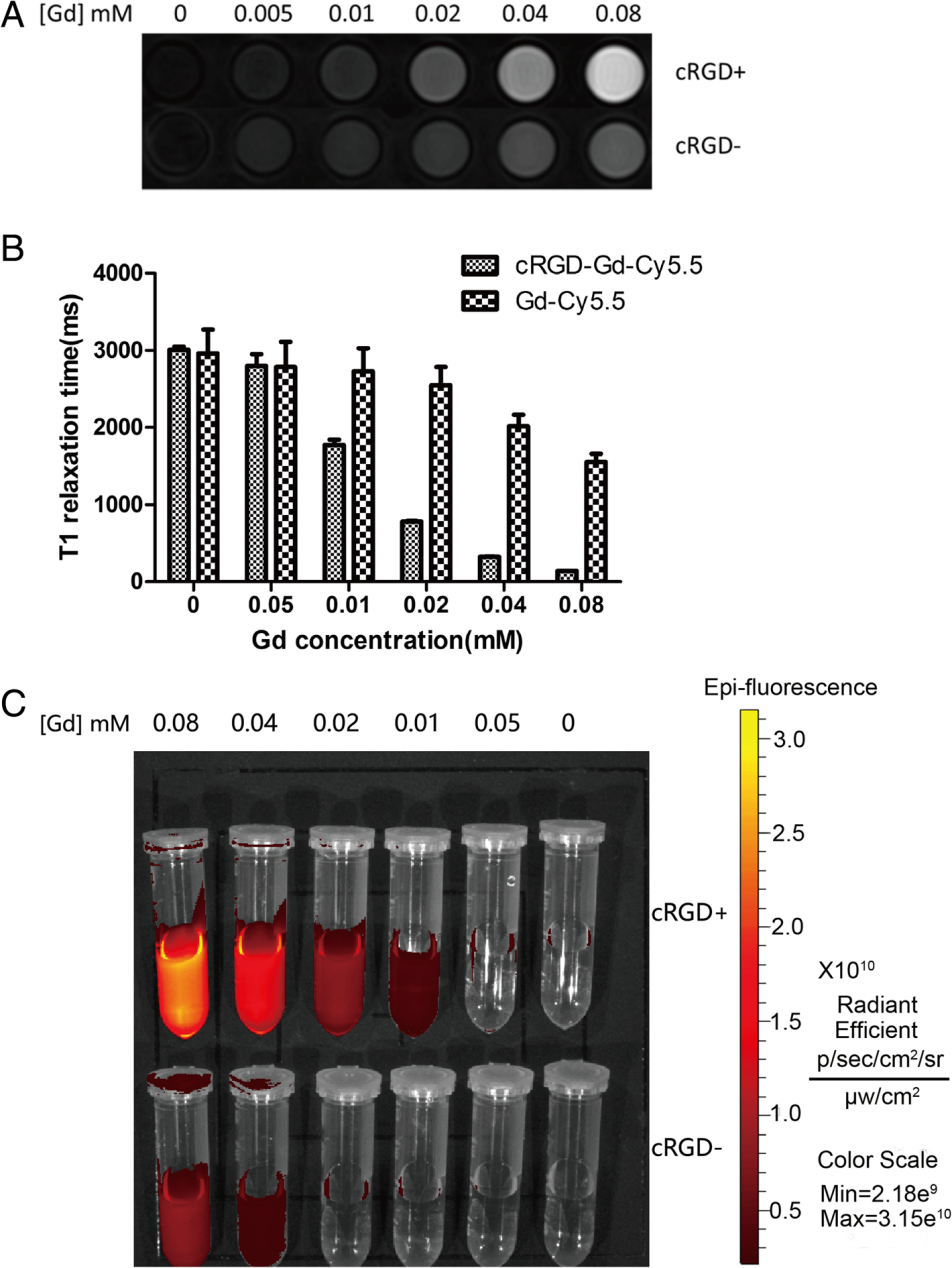
Cell magnetic resonance and fluorescence imaging. **a** The cells were incubated with A549 cells for 2 h according to the Gd concentrations: 0, 0.005, 0.01, 0.02, 0.04, and 0.08 (mM). The Gd-Cy5.5 was used as the control group. **b** T1 relaxation time of the corresponding magnetic resonance image. **c** Small animal live imaging system images

Based on its high r1 relaxation rate and good cell compatibility, cRGD-Gd-Cy5.5 was used as a positive contrast agent for MR imaging of cancer cells in vitro. As shown in Fig. 6a, b, the color of the T1-weighted MR images gradually became brighter, indicating that the MR signal intensity of A549 cells treated with cRGD-Gd-Cy5.5 increased with higher Gd concentrations. The difference in the T1 relaxation time between the two groups was statistically significant, as indicated by the *t* test for two independent samples (*p* < 0.05). These data suggest that synthetic cRGD-Gd-Cy5.5 has the potential for use as a positive contrast agent in in vitro MR imaging of cancer cells. Similarly, a small animal fluorescence system showed a corresponding increase in fluorescence intensity as the probe concentration increased (Fig. 6c). From the image signal and the specific data, the imaging effect of the target group (cRGD-Gd-Cy5.5) was better than that of the non-targeting group (Gd-Cy5.5).

### In Vivo MR and Fluorescence Imaging

**Fig. 7:**
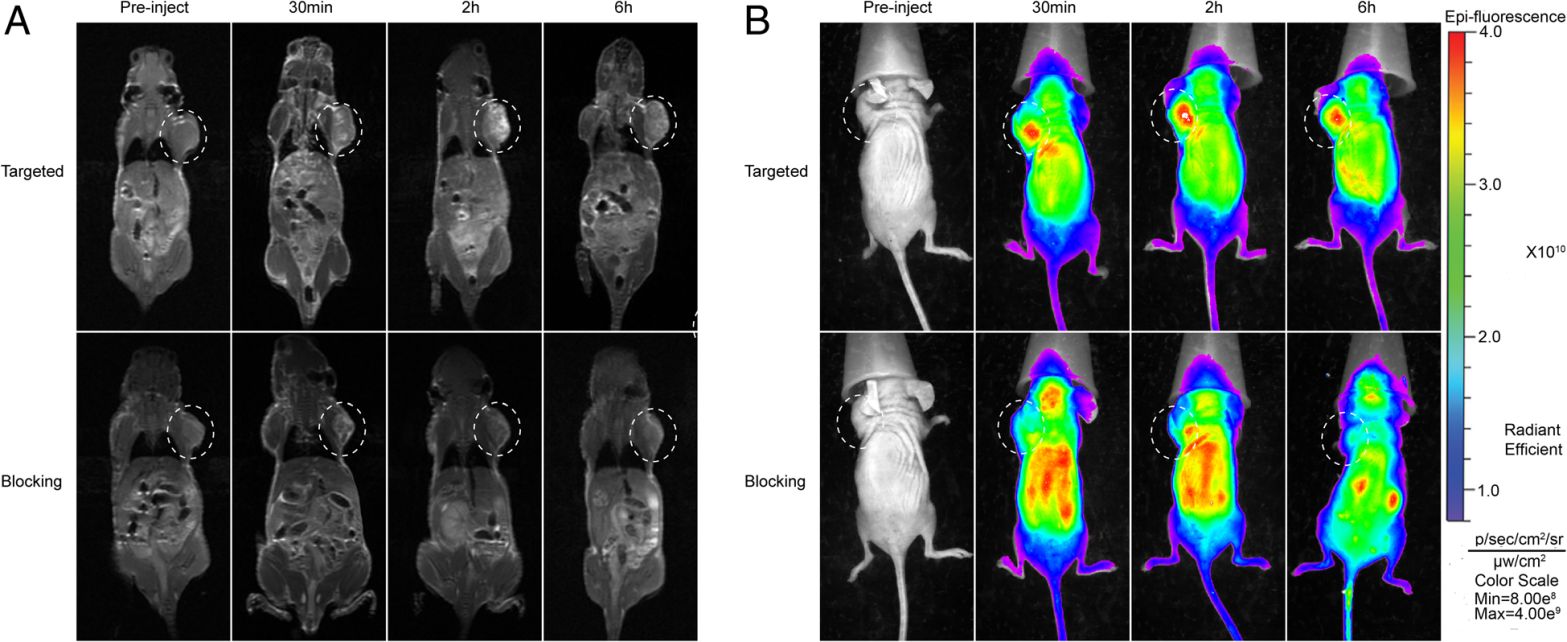
MR and fluorescence imaging of nude mice nasopharyngeal carcinoma subcutaneous xenografting tumor models. **a** Nude mice were anesthetized and imaged with a 3.0-T MRI scanner at preinjection and at 0.5, 2, and 6 h postinjection. **b** Nude mice were anesthetized and imaged with fluorescence imaging system at 0.5, 2, and 6 h postinjection

The MRI images acquired before injection of contrast agents showed no significant signal differences between tumors and other peripheral organs/tissues. At 5 min after injection with CRGD-Gd-Cy5.5, the signal intensity at the tumor sites started to be strengthened, but stable hyperintensity was maintained since 6 h after the injection. In the test group, intensified tumor parenchyma was observed since 30 min, and the reinforcement was enhanced at 2 h (Fig. 7a). In the receptor-blocked group, however, only gentle reinforcement was found at the margins of tumors, and the reinforcement degree was weakened at 2 h and already disappeared (Fig. 7a). On the fluorescence images, the fluorescence intensity in the targeted group gradually enhanced since the 30th minute, but was still high at the sixth hour (Fig. 7b), indicating the blood circulation was extended and CRGD-Gd-Cy5.5 efficiently concentrated in tumors. In the receptor-blocked group, no specific probe concentration was observed at any of the tested time points. High-intensity fluorescence at the bilateral kidney was observed at the sixth hour (Fig. 7b). We deduce that the metabolic pathway of CRGD-Gd-Cy5.5 may be the clearance through the kidney system.

## Discussion

Integrin αvβ3 is highly expressed not only in the endothelial cells of the tumor neovasculature but also on the surface of a variety of tumor cells, such as malignant melanoma, malignant glioma, prostate cancer, lung cancer, and breast cancer cells [15], while αvβ3 is not expressed or expressed at low levels in regular epithelial cells and mature vascular endothelial cells. Based on the above properties, integrin αvβ3 has become an ideal target for in vivo monitoring of tumor metastasis [16]. However, the current methods of measuring integrin αvβ3 expression levels still have issues of noninvasiveness, repeatability, and imaging timeliness, which remain to be solved. In view of the above objective issues, a dual-mode molecular probe was synthesized in this study for real-time, dynamic monitoring of integrin αvβ3 expression, which indirectly achieved the goal of predicting and monitoring tumor metastasis.

Contrast agents can be divided into two types according to the mechanism of MR imaging: Gd compounds (T1-positive contrast agent) and paramagnetic iron oxide nanoparticles (T2-negative contrast agent). Compared with other contrast agents, Gd compounds have unparalleled advantages in clinical practice [17]. The Gd ion, which is a superparamagnetic material, provides a high-intensity signal in T1-weighted images. Gd can also bind to a variety of substances to form stable complexes with a high spatial resolution and a high signal-to-noise ratio. However, each type of contrast agent has its own limitations (namely, the short blood circulation time of T1 contrast agents and the magnetic susceptibility artifacts of T2 contrast agents) [18–21]. Traditional Gd compounds have the following limitations. (1) Traditional Gd chelates have no targeting effect. (2) The signal intensity of Gd chelate contrast agents is lower than that of ultrasmall superparamagnetic iron oxide particles (USPIOs) at the same concentration. To solve the above issues, Gd ions are linked to macromolecular structures (such as liposomes, dendritic molecules, and dextran) to enhance the T1 relaxation effect [22], and the complex is then linked to the ligand of tumor targets to prepare molecular probes and achieve tumor-targeted imaging [23]. In this study, liposomes were selected as the carrier molecule that links MR contrast agents to fluorescent contrast agents. The special spatial structure of liposomes has a biological signal amplification effect, and it effectively improves the concentration of Gd ions in local tissues, thereby enhancing the MR signal intensity. Zhou et al. [24] employed β-cyclodextrin-linked polyhedral oligomeric silsesquioxane (POSS) nanoparticles as the carrier to couple RGD to Gd compounds to successfully obtain a molecular probe, cRGD-POSS-βCD-(DOTA-Gd), with a T1 relaxation rate of 9.50 mM^−1^ S^−1^. The liposome-cRGD-Gd-Cy5 complex obtained in this study was found to have a T1 relaxation rate of 10.515 mM^−1^ S^−1^, which is more than twice the T1 relaxation rate of the Gd agent currently in clinical use (Magnevist), and this complex also exhibited excellent MR imaging properties.

The nanoprobe prepared by our group has a uniform size, a regular appearance, and good dispersibility. The zeta potential reflects the stability of the molecular probe, and a higher positive or negative value of the zeta potential is associated with a more stable system and less possibility of aggregation. In general, molecules with zeta potentials higher than + 30 mV or lower than − 30 mV are considered to have good stability. The zeta potential on the surface of the probe particle obtained in this study was + 39.5 ± 1.65 mV, which is slightly lower than + 30 mV. Nevertheless, no aggregation was observed under TEM.

Gd agents are the most widely used MR contrast agent in current clinical practice, and their biosafety cannot be ignored. Guo et al. [25] found that a Gd complex coupled to dendritic hyaluronic acid was a very safe and effective microparticle as an MR contrast agent, with a high sensitivity and a low residual presence in the human body. Through the study of the in vitro cytotoxicity of the constructed molecular probe, this molecular probe was demonstrated to have low cytotoxicity and high biosafety for tumor cells as well as non-tumor cells (epithelial cells and vascular endothelial cells). We believe that liposomes have good biocompatibility, and the low biotoxicity may be a benefit of the liposome encapsulating the Gd ions.

Based on previous studies [26–28], our group selected the A549 lung adenocarcinoma cell line with high expression of integrin αvβ3 for use in the experimental group, with the innovative addition of the SUNE-1-5-8F nasopharyngeal carcinoma cell line in the in vitro cell-targeting experiments; mammary epithelial cells with nearly no expression of integrin αvβ3 were included as the negative control group. The molecular probe bound well to the cell membrane of the A549 lung adenocarcinoma cells and the SUNE-1-5-8F nasopharyngeal carcinoma cells but did not bind to MCF-10A cells, indicating that the probe has an excellent molecular-targeting ability, which was confirmed by the results of the immunofluorescence assay. MR imaging results further confirmed the targeting property of the liposomal cRGD-Gd-Cy5.5 molecular probe in MR, with signals of higher intensity than that of non-targeted contrast agent (Gd-Cy5.5) in tumor cells. In vivo experiments confirm that probes can maintain stable in the serum and persistently target the tumor sites for 6 h at least. The results from the in vitro studies ensure subsequent in vivo studies of the molecular probe in a metastatic model of nasopharyngeal carcinoma in nude mice.

## Conclusions

In summary, the preparation method for the dual-mode probe cRGD-Gd-Cy5.5 targeting integrin αvβ3 is feasible, and this probe has desirable stability and biosafety and a high T1 relaxation rate. The probe showed a strong targeting effect toward the cells with high integrin αvβ3 expression, which laid a solid foundation for noninvasive, efficient, and real-time dynamic monitoring of tumor metastasis at the anatomical level and the molecular metabolic level via MR/fluorescence molecular imaging in vivo.

## Methods

### Synthesis of cRGD-Gd-Cy5.5

To 20 mL of chloroform, 70 mg of lecithin, 20 mg of cholesterol, and 210 mg of DSPE-PEG2000-NH were added, and the mixture was placed in an ultrasonic cleaner to completely dissolve the substances (as indicated by a clear solution without granular substances). The solution was then placed in a pear-shaped flask for rotary evaporation in a 60 °C water bath until a honeycomb-like film (no liquid residue) was formed. Gd trichloride hexahydrate (10 mg) was precisely weighed and dissolved in carbonate buffer at pH 8.5 to obtain a clear solution, which was then mixed with the above honeycomb-like film for hydration at 50 °C for 1 h; this step was followed by dispersion and refinement through an ultrasonic probe in an ultrasonic liquid processor (VCX 750, Sonics, USA). Finally, the mixture was collected and passed through a 0.22-μm filter and subjected to ultrafiltration with a 10-kD ultrafiltration tube to remove free Gd trichloride and obtain liposomes carrying Gd trichloride, which were then stored at 4 °C.

The RGD cyclic peptide was then coupled to the fluorescent Cy5.5 molecule. RGD cyclic peptide (5 mg) was dissolved in 1 mL of dilute hydrochloric acid buffer at pH 5.0, and 1 mg of 1-ethyl-3-(3-dimethylaminopropyl)carbodiimide hydrochloride (EDC) and 0.5 mg of *N*-hydroxysuccinimide (NHS) were added and then activated at room temperature for 30 min in an oscillator at constant temperature. The activated RGD cyclic peptide molecule was then mixed with 100 μg of the Cy5.5 fluorescent molecule and the liposome, and the pH was measured as 8.4 using precision pH test paper. The mixture was then incubated at room temperature in a shaker for 4 h; this step was followed by the removal of unreacted RGD cyclic peptide and fluorescent Cy5.5 molecule through the use of a 10-kD ultrafiltration tube.

### Characterization of Nanoparticles

The particle size of the nanoparticles was determined by TEM (GEOL Tokyo, Japan). A small amount of liposome-cRGD-Gd-Cy5.5 complex was added to ultrapure water (pH = 6.0) for dilution into a 1 mg/mL solution. A drop of the sample was placed on a coated copper grid by using a sterile transfer pipette, and 2% sodium phosphotungstate was added dropwise after a few minutes for restaining. Excessive negative staining solution was aspirated after 1–2 min. The copper grid was then dried and placed under an 80-kV transmission electron microscope for observation. Approximately 40 to 50 nanoparticles were randomly selected to observe their morphology and particle size, and images of 50-nm and 100-nm scale were saved. The particle size was measured using the NanoMeasure software, and the average was calculated from three repeated measurements.

A particle size analyzer (Malvern, UK) was used to measure the hydrodynamic size and zeta potential of nanoparticles. A drop of liposome-cRGD-Gd-Cy5.5 complex solution was diluted with ultrapure water and then transferred to an Eppendorf tube, which was placed in an ultrasonic cleaner for 5 min to disperse the particles evenly. The dispersed particles were evaluated by DLS in the nanoparticle size analyzer to determine the particle size and zeta potential.

The optical properties of the liposomal cRGD-Gd-Cy5.5 complex were characterized using a multifunctional microplate reader (BioTek, USA). Two microliters each of liposome solution and liposome-cRGD-Gd-Cy5.5 was added to 998 μL of phosphate-buffered saline (PBS) buffer and mixed well before being added to a quartz cuvette; the ultraviolet absorption at 400–800 nm was then obtained via a multifunction microplate reader. The liposome-cRGD-Gd-Cy5.5 complex (1.5 mL) solution was transferred to an Eppendorf tube, and the same amount of empty liposome solution was used as a blank control. Fluorescence imaging was carried out using a small animal fluorescence imaging system.

The modification of surface amino groups on liposomes was confirmed using FT-IR spectroscopy (Nicolet-5700, USA). The UV–vis absorption spectrum was obtained (UV 2550, Shimadzu, Japan); a concentration of 0.1 mg/mL liposomal cRGD-Gd-Cy5.5 was used for the measurement.

### Measurement of the Relaxation Rate

The cRGD-modified Gd-loaded liposomal fluorescence probe sample was diluted with deionized water to obtain the following Gd concentrations: 0.18, 0.1275, 0.085, 0.0425, and 0.017 mM. Five milliliters of each diluted sample was placed in a vial with a screw cap, and the vials were then fixed on a multifunctional tube rack according to the order of the concentration. The cRGD-modified Gd-loaded liposomal fluorescent probe sample underwent MR scanning through the head and neck coils to obtain T1-weighted images for the different concentrations of the probe. The scan sequence used for the T1-weighted images was a modified look-locker inversion recovery (MOLLI) sequence, where the parameters were set as follows: repetition time (TR) = 5.8 ms, echo time (TE) = 3.66 ms, inversion recovery time (TI) = 16–3200 ms, and scanning thickness = 5 mm. After scanning was completed, a pseudo-color map was obtained. A 0.3-cm^2^ area of interest was delineated in the image of each sample in the map, and the corresponding T1 value was read.

### Cell Culture and Cytotoxicity Assay

A549 human lung adenocarcinoma cells, SUNE-1-5-8F human nasopharyngeal carcinoma cells, human umbilical vein endothelial cells (HUVECs), and MCF-10A human breast epithelial cells were obtained from the American Type Culture Collection (ATCC, Manassas, VA). The cells were cultured in complete 1640 medium (Euroclone-Lonza) containing 10% fetal bovine serum (Euroclone-Lonza), 100 units/mL penicillin, 100 μg/mL streptomycin, and 2 mM glutamine under 5% CO_2_ at 37 °C.

The CCK-8 method was used to examine the cytotoxicity of the molecular probe to different cells, including normal epithelial cells and tumor cells. A549 cells, 5-8F cells, HUVECs, and MCF-10A cells were seeded in a 96-well plate at a density of 5 × 10^3^ cells/well and cultured overnight. The adherent cells were then incubated with 100 μL of fresh 1640 medium containing cRGD-Gd-Cy5.5 of various Gd concentrations (0, 50, 100, 200, and 400 μM) for 24 h. Subsequently, the cells were washed three times with PBS and then incubated with 100 μL of Dulbecco’s modified Eagle medium (DMEM) containing no fetal bovine serum (FBS). After 10% Cell Counting Kit-8 reagent (Dojindo, Japan) was added to each well, the sample was incubated for 10 h. The absorbance at 450 nm of each well was then measured using an ELISA microplate reader (Multiskan MK3, Thermo Scientific, Logan, UT). Cells treated with PBS only were employed as the control group. For each sample, five parallel wells were analyzed to obtain the mean and standard deviation.

### Immunofluorescence Staining and Flow Cytometry Assay of Integrin αvβ3

A total of 20,000 A549, 5-8F, and MCF-10A cells each were seeded in confocal plates (Thermo Scientific) and cultured for approximately 24 h when the cells successfully grew on the slides. The cells were washed three times with PBS, fixed with 4% paraformaldehyde (Boster) at room temperature for 30 min, and then washed three times (10 min each) in PBS. The cells were then incubated in 3% bovine serum albumin (PBST) for 30 min to block non-specific antibody binding and washed three times with PBS. The fixed cells were incubated with anti-integrin αvβ3 monoclonal antibody (1:500; Abcam, Cambridge, UK) at 4 °C overnight and then incubated with anti-mouse fluorescent secondary antibody (1:500) (Abcam) for 1 h. 4′,6-Diamidino-2-phenylindole (DAPI; 1:1000; Sigma) was used to stain the nucleus. A laser confocal scanning microscope (Nikon A1, Tokyo, Japan) was used to detect the green fluorescence signal from integrin αvβ3 and the DAPI signal from nuclei.

The integrin αvβ3 expression levels in different cell lines were quantified using flow cytometry. In brief, three types of cells were collected: A549, SUNE-1-5-8F, and MCF-10A, which were washed in PBS containing 1% bovine serum albumin (BSA). After being sealed with the PBS containing 5% BSA, the cells were cultivated in VNR-1 antibody (Abcam, Cambridge, MA, USA) at density 2 μg/1 × 10^6^ and 4 °C for 30 min. The secondary antibody was DyLight 488 goat anti-mouse IgG (H+L) (ab96879), diluted 1/500, and at 22 °C for 30 min. Quantitative assays were conducted on a flow cytometer (Gallios, Beckman Coulter, USA) at excitation wavelength 488 nm and emission wavelength 530 nm. Each time, at least 1 × 10^5^ cells (*n* = 3) were collected.

### Binding Assay of the Molecular Probe to Integrin αvβ3

A549, SUNE-1-5-8F, and MCF-10A cells were inoculated into confocal plates. The cells were incubated at 37 °C for 1 h with the molecular probe of the same Gd concentration after the cells reached the logarithmic growth phase. The cells were then washed three times in PBS and placed under a laser confocal scanning microscope for observation.

### In Vitro MR Imaging and Fluorescence Imaging of Tumor Cells

To verify the tumor cell-targeting ability of the cRGD-modified Gd-loaded liposomal fluorescent probe, the probe was incubated with A549 cells and then examined using MR imaging and a small animal fluorescence imaging system. A549 cells were seeded in six-well plates with 2 mL of 1640 medium containing 10% FBS and 1% penicillin–streptomycin in each well and then incubated at 37 °C and 5% CO_2_. In the experimental groups, cRGD-Gd-Cy5.5 was incubated with A549 cells for 2 h with Gd concentrations of 0, 0.005, 0.01, 0.02, 0.04, and 0.08 mM. The cells of the control groups were incubated with the non-targeted gadolinium contrast agent (Gd-Cy5.5) for 2 h at the same Gd concentrations as the experimental groups. The cells were then washed with PBS, trypsinized, centrifuged, and finally placed in glass tubes containing 1 mL of PBS (with 0.5% agarose) for MR imaging. Specific imaging parameters were described above. A small animal live imaging system (IVIS Lumina XRMS Series III, MA, USA) was used for fluorescence imaging of the A549 cells.

### In Vivo MR and Fluorescence Imaging

To validate whether CRGD-Gd-Cy5.5 had tumor targetability in vivo in animals, we conducted animal experiments following the Guide for Care and Use of Laboratory Animals, released by the Animals Ethics Committee of Laboratory Animal Center, Guangxi Medical University. We successfully established a Balb/c nude mice nasopharyngeal carcinoma subcutaneous xenografting tumor model for magnetic resonance/fluorescent scanning. A 3.0-T MRI scanner containing 40-mm-diameter mouse volume coils (Discovery 750, GE, Germany) was used for T1-weighted imaging, operated at field of view = 80 × 80 mm, repetition time = 742 ms, echo time = 69 ms, and layer thickness = 2.0 mm. The nude mice were divided into two groups (*n* = 3), including a test group and a receptor-blocked group. All mice were injected via tail vein with CRGD-Gd-Cy5.5 (0.05 mmol Gd/kg). Each mouse was scanned at four time points: before injection, 30 min, 2 h, and 6 h after injection. In the receptor-blocked group, 1 h before injection with CRGD-Gd-Cy5.5, each mouse was injected via the tail vein with free CRGD polypeptide (10 mg) and was MRI-scanned at the same four time points. The fluorescent images were photographed by a fluorescence imaging system (Bruker, USA). The grouping, drug injection, and scanning time points were all the same as described above. During the imaging, the mice (*n* = 3) were anesthetized using a gas mixture of oxygen and isoflurane. The nude mice were kept at heart rate 60–120/min and respiratory rate at 20–40/min. Finally, direct visual comparison of tumor images was conducted by two experienced radiologists.

## Abbreviations

DLS: Dynamic light scattering
DMEM: Dulbecco’s modified Eagle medium
ECM: Extracellular matrix
EDC: 1-Ethyl-3-(3-dimethylaminopropyl)carbodiimide hydrochloride
FBS: Containing no fetal bovine serum
HUVECs: Human umbilical vein endothelial cells
MOLLI: Modified look-locker inversion
MR: Magnetic resonance
NHS: *N*-Hydroxysuccinimide
PEG: Polyethylene glycol
RGD: Arginine–glycine–aspartate
TE: Echo time
TEM: Transmission electron microscopy
TI: Inversion recovery time
TR: Repetition time
USPIOs: Iron oxide particles
